# National action plans on antimicrobial resistance in Latin America: an analysis via a governance framework

**DOI:** 10.1093/heapol/czad118

**Published:** 2024-01-05

**Authors:** Paula Avello, Lisa M Collins, Sonia A Gómez, Federico Luna, Mariano E Fernández Miyakawa, Helen M West, Graziella Iossa

**Affiliations:** Faculty of Biological Sciences, School of Biology, University of Leeds, Woodhouse, Leeds LS2 9JT, UK; Faculty of Biological Sciences, School of Biology, University of Leeds, Woodhouse, Leeds LS2 9JT, UK; Servicio Antimicrobianos, INEI—ANLIS ‘Dr. Carlos G. Malbrán’, Buenos Aires CP1281, Argentina; Servicio Nacional de Sanidad y Calidad Agroalimentaria (SENASA), Buenos Aires CP1063, Argentina; Instituto Nacional de Tecnología Agropecuaria (INTA/CONICET), Buenos Aires CP1686, Argentina; School of Biosciences, University of Nottingham, Sutton Bonington Campus, Loughborough LE12 5RD, UK; School of Life and Environmental Sciences, University of Lincoln, Lincoln LN6 7TS, UK

**Keywords:** Antimicrobial resistance, national action plans, global action plan, governance

## Abstract

In 2015, the World Health Assembly adopted a global action plan (GAP) on antimicrobial resistance (AMR). Member states were encouraged to develop their own national action plans (NAPs) in alignment with the GAP. To-date, in systematic assessments of NAPs, the Latin American specific context has not been previously analysed. Here we examined 11 Latin American NAPs published between 2015 and 2021 using content analysis. We focused on two approaches: (1) alignment between the strategic objectives and actions defined in the GAP, and those outlined in the NAPs via a content indicator; and (2) assessment of the NAPs via a governance framework covering ‘policy design’, ‘implementation tools’ and ‘monitoring and evaluation’ areas. We observed a high alignment with the strategic objectives of the GAP; however, the opposite was observed for the corresponding actions. Our results showed that the governance aspects contained within coordination and participation domains were addressed by every Latin American NAP, whereas monitoring and assessment areas, as well as incorporating the environment, would need more attention in subsequent NAPs. Given that AMR is a global health threat and collective efforts across regions are necessary to combat it, our findings can benefit member states by highlighting how to strengthen the AMR strategies in Latin America, while also supporting global policy formulation.

Key messages

- We analysed national action plan (NAP) documents on antimicrobial resistance from 11 Latin American countries using content analysis.
- The strategic objectives of the NAPs in Latin America are aligned with those of the global action plan on antimicrobial resistance.
- Many governance aspects were addressed by all NAPs, including coordination, participation and equity domains.
- Aspects of monitoring and evaluation, and activities in the environment sector, would need more attention in subsequent NAPs.

## Introduction

The use of antimicrobials has played a key role in reducing morbidity and mortality from microbial infections in humans, plants and animals. Today they are a key underpinning feature of modern medicine (Hutchings *et al*., 2019). However, the overuse of antimicrobials has accelerated the evolution of resistance in microorganisms (Laxminarayan *et al*., 2013; O’Neill, 2016; Hutchings *et al*., 2019); consequently, antimicrobial resistance (AMR) has become a global health threat (Laxminarayan *et al*., 2013). In 2019, global estimates attributed 1.27 million deaths to bacterial AMR (Murray *et al*., 2022). This death burden is not uniformly spatially distributed. The number of deaths and death rates (deaths per 100 000 population) attributable to AMR, as well as the proportion of deaths accountable to pathogens and pathogen–drug combinations associated with resistance, vary considerably by region, e.g. from a death rate of 6.5 in Australasia to 18.6 in Southern Latin America (Murray *et al*., 2022). Moreover, resistance of different pathogens to antimicrobials is a region-dependent phenomenon (Prestinaci *et al*., 2015). Whilst the need for unified global action is clear, these geo-spatial disparities highlight the necessity for efforts to be regionalized. With over two in five infection-related deaths associated with AMR in the Americas in 2019 (Aguilar *et al*., 2023), the need for analysis of the Latin American context is urgent.

The World Health Assembly in 2015 adopted a global action plan (GAP) on antimicrobial resistance centred on the ‘One Health’ approach (World Health Organization, 2015a). This approach recognises the importance of collaborative work among human, animal and environment sectors to address public health threats (World Health Organization, 2015b). Recommended actions, corresponding to the five strategic objectives of the GAP (World Health Organization, 2015b), encompass interventions in human health, animal health—including food animals—and agriculture sectors. Member States agreed to develop a national action plan (NAP) aligned with the GAP to improve their plans and actions in stages from 2015, and to provide annual progress reports on the implementation of the NAPs via the tripartite AMR country self-assessment survey (TrACSS) (World Health Organization, 2020a). Self-reported engagement with the NAP appears to be related to individual countries’ efforts to tackle AMR. A recent study showed an inverse relationship between progress towards a NAP and AMR mortality rates in the Americas, with the countries that had the lowest mortality rates also showing greater progress in their AMR NAPs (Aguilar *et al*., 2023).

Several systematic content analyses have assessed member states policies on AMR using exclusively the NAP documents. In 2021, NAPs from Southeast Asia (Chua *et al*., 2021) were analysed using the governance framework on AMR proposed by Anderson *et al*. (2019). This framework synthesises documented international guidance as well as the opinion of experts from government departments, international organizations, academia and policy institutes. It allows both assisting evaluation of NAPs, and helping policymakers to create and improve NAPs. Further work used a content indicator to measure the alignment between the NAPs and the GAP by calculating the frequency of key terms associated with the objectives of the GAP and their actions (Munkholm and Rubin, 2020), or a policy framework for actors, context and content (Willemsen *et al*., 2022). In both cases, Latin American NAPs were excluded from analyses due to the documents being published in Spanish and Portuguese. A recent systematic governance analysis of 114 countries expanded the searches beyond the NAP documents to evaluate the response to AMR (Patel *et al*., 2023), analysing mostly implementation aspects of the countries’ strategies. Machine translation was used for non-English documents (Patel *et al*., 2023).

In this study, our approach differs. We manually translated and analysed all NAPs on AMR available between 2015 and 2021 from Latin America to examine the countries’ strategy design against AMR in the region (Table 1). We carried out a systematic content analysis assessment and centred it on a governance framework review exclusively on NAP documents, with the aim to highlight the specific way Latin American countries are framing AMR policies and how the Latin American policies compare with countries in other regions. We asked whether (1) Latin American action plans were aligned with the GAP agreed in 2015 and (2) whether there are Latin American context-specific needs where efforts should be focused on improvement of future NAPs. Strengths and weakness of the documents’ contents are given for consideration in the subsequent Latin American NAPs, both for new iterations of existing plans and as a baseline for those countries who have not released their plans yet. Governance aspects in common with other regions that need to be strengthened and the inclusion of the One Health approach are also considered in the analysis.

**Table 1.** Duration period of NAPs on AMR in Latin American countries

Country^a^	Year of publication	Ending of the NAP period	Number of pages	World Bank Classification	NAP status for publication^b^	Included in analysis (Yes/No)
Argentina	2015	NM	28	UMIC	Approved	Yes
Brazil	2018	2022	25	UMIC	Approved	Yes
Chile	2017	NM	43	HIC	Approved	Yes
Costa Rica	2018	2025	36	UMIC	Approved	Yes
Colombia	2018	NM	66	UMIC	Approved	Yes
Ecuador	2019	NM	38	UMIC	Approved	Yes
Mexico	2018	NM	31	UMIC	Approved	Yes
Nicaragua	2020	2024	20	LMIC	Approved	Yes
Paraguay	2018	2020	40	UMIC	Approved	Yes
Peru	2017	2021	96	UMIC	Approved	Yes
Uruguay	2018	NM	35	HIC	Approved	Yes
Cuba	–	–	–	UMIC	In development	No
Dominican Republic	–	–	–	UMIC	In development	No
Honduras	–	–	–	LMIC	In development	No
Panama	–	–	–	HIC	Not yet approved	No
Bolivia	–	–	–	LMIC	Not yet approved	No

a All NAPs were published in Spanish, except the NAP from Brazil, which was published in Portuguese.

b By the time of publication of the Tripartite AMR Country Self-assessment Survey 2020–2021.

NM = not mentioned.

## Methods

### Approach to content analysis

A content analysis was carried out based on two methods: (1) using a content indicator proposed by Munkholm and Rubin (2020) to assess alignment between the strategic objectives and actions defined in the GAP and those outlined in the NAPs; and (2) following the structure of an AMR governance framework proposed by Anderson *et al*. (2019) to assess specific components of the governance areas ‘policy design’, ‘implementation tools’ and ‘monitoring and evaluation’. Additionally, we scored the NAPs according to the framework indicators (Table 2). The scores were summarised in frequency plots showing the percentage of indicators addressed in the NAPs to provide an overview of Latin American policies on AMR.

**Table 2.** The governance framework developed by Anderson *et al*. (2019)

Area	Domains	Indicators
Policy design	Strategic vision	1. Has situational analysis been done to determine the prevalence and incidence of AMR organisms in the country?
		2. Is a NAP in place, if not what is the timeframe for developing and implementing the NAP?
		3. Are the objectives contained within the NAP specific, measurable and time-bound?
		4. Are there quantitative targets for AMR or antimicrobial use outlined in the NAP?
	Coordination	5. Is coordination between sectors and across different levels of each sector considered?
		6. Is there a ministry or intersectoral committee, or both, responsible for coordination and implementation?
	Participation	7. Was a high level of stakeholder participation facilitated throughout the development of the NAP?
		8. Are the activities in the NAP inclusive across all sectors related to One Health? If so, how, and if not, why not?
		9. Was there support from a technical advisory group or subject matter experts during development of the NAP?
	Accountability	10. Is there a ministry or intersectoral committee, or both, responsible for coordination and implementation that is accountable to the government?
		11. Is a responsible person nominated in each sector and do agreements exist regarding what happens if objectives are not met?
	Transparency	12. Is the complete NAP publicly available?
		13. Are all progress reports publicly available?
		14. Is all funding information publicly available?
		15. Is all AMR and antimicrobial use surveillance data publicly available?
	Sustainability	16. Is there either a written mandate or voluntary agreement from all relevant sectors in place to implement the NAP?
		17. Are there dedicated budgets in place to implement specific activities in the NAP?
		18. Is there an assessment of future budget requirements for different activities listed in the NAP?
		19. Is there ongoing support from a technical advisory group or subject matter experts during implementation, monitoring and evaluation of the NAP?
	Equity	20. Does the NAP include both encouraging responsible use and facilitating equitable access to existing essential antimicrobials?
Implementation tools	Surveillance	21. Is there a national surveillance system for resistant organisms across the human, animal and the environmental health sectors?
		22. Is there a national surveillance system for levels of antimicrobial use in animals and humans?
		23. Is there adequate laboratory capacity and capability supported by regular external quality assessments?
	Antimicrobial stewardship	24. Are there stewardship programmes across human and animal health sectors?
		25. Are rapid diagnostic tools widely available and in regular use?
		26. If so, do national guidelines regarding their indication and interpretation exist?
		27. Is there any use of financial and non-financial incentives or penalties in animal and human health to reduce inappropriate use of antibiotics?
	Infection prevention and control	28. Are there IPC policies across all levels of human, animal and environmental health sectors?
		29. Are there up-to-date national guidelines for IPC across human, animal and environmental health sectors?
		30. Are immunization programmes used as an approach to prevent infections across human and animal health sectors?
		31. Are financial and non-financial incentives or penalties for IPC policies used across human, animal and environmental health?
	Education	32. Are there certifications or programmes in place to ensure a basic education for all involved groups of professionals to deliver necessary understanding for strategies to tackle AMR?
		33. Are there continuing education programmes for all involved groups of professionals to ensure expertise necessary for expanding knowledge and sustained efforts to tackle AMR?
		34. Is there a workforce strategy that aims to deliver the sustainable supply of the necessary workforce required to deliver antimicrobial stewardship and IPC policies?
	Public awareness	35. Are there multimodal public awareness campaigns that focus on AMR and educational programmes (including school children) related to AMR?
		36. Do the implemented public awareness campaigns have an ongoing character?
		37. Does the conception of the public awareness campaign consider aspects of behavioural sciences, social science and psychology?
	Medicines regulation	38. Are there regulations in place to ensure appropriate use of antimicrobials in human health?
		39. Are there regulations in place to ensure appropriate use of antimicrobials in animal health?
		40. Is there an authority in place to monitor and enforce legislation, if so does this authority have a dedicated budget?
	Fostering R&D and facilitating market access to novel products	41. Is fostering research and development and facilitating market access to novel antimicrobials, diagnostics, vaccines and alternative treatments in both human and animal health listed as a priority in the NAP?
		42. Does the NAP consider how the country can contribute to research and development of novel agents at both a national and international level?
		43. Is there a dedicated national budget for research and development of novel antimicrobials, diagnostics, vaccines or alternative treatments?
Monitoring and evaluation	Reporting	44. Are annual AMR NAPs progress reports published?
		45. Are annual surveillance reports published containing data regarding the incidence of resistant organisms and antimicrobial use?
		46. Is there collaboration with and systematic data transmission to international surveillance systems?
	Feedback mechanisms	47. Are there feedback mechanisms in place that relay surveillance data back at both regional and organizational levels?
		48. Are there regular deadlines in place to review progress of specific actions within the NAP, and arrangements to feedback at both regional and organization levels?
	Effectiveness	49. Have there been efforts to evaluate the effectiveness (e.g. measure of effect on human and animal health) of specific policies or interventions, or both, implemented?
		50. Have efforts been made to evaluate the cost-effectiveness (e.g. measure of effect on human and animal health) of specific policies or interventions implemented?
	AMR research	51. Is research to understand both the drivers and effects of AMR and potential policies and interventions identified as a key priority in the NAP?
		52. Is there a dedicated national budget for AMR research in place?

### Data collection and sources

A total of 11 NAP documents (Table 1) were obtained (on 31 May 2021) from the WHO AMR library (World Health Organization, 2020b) and governmental websites by the time of publication of the TrACSS 2020–2021 (Government of Argentina, 2015; Government of Chile, 2017; Government of Peru, 2017; Government of Brazil, 2018; Government of Colombia, 2018; Government of Costa Rica, 2018; Government of Mexico, 2018; Government of Paraguay, 2018; Government of Uruguay, 2018; Government of Ecuador, 2019; Government of Nicaragua, 2020). NAPs were reviewed manually by one person repeatedly (a minimum of three times) analysing the content of the documents. Content analysis and scoring (see below) were then discussed by a minimum of two authors (PA and GI). NVivo 12 software (QSR International Pty Ltd, 2018) was used to find occurrences of specific text. The non-text format NAPs from Chile and Ecuador were reviewed without the support of any text search software.

The analysis was based on the published documents and did not consider any additional information from external sources—such as governmental websites, or other policies—with the exception of indicators 12 and 44 (see Table 2) as both questions can be intrinsically answered from the WHO website [Library (World Health Organization, 2020a) and TrACSS (World Health Organization, 2020b) sections]. The NAPs were manually translated by one of the project team who is a native Spanish speaker with fluent knowledge of English (PA).

### Data analysis

#### Method 1: the content indicator of Munkholm and Rubin (2020)

To assess representativeness of the five strategic objectives defined in the GAP and the corresponding actions, key terms (see supplementary Table S1 in the online supplementary material) were searched in the NAPs that related to each of the five objectives and the corresponding actions. Using the Text Search query tool in NVivo 12, the terms became query items and their occurrence in each Latin American NAP was recorded to allow a comparison between the Latin American countries with countries from other regions. The same 51 Text Search query items used in Munkholm and Rubin (2020) were translated into Spanish (12 query items for the strategic objectives and 39 for the actions, Table S1). To avoid missing those terms that cannot be found in Spanish due to semantics and grammatical use, we defined new terms (see Table S1). All terms were present in the Spanish version of the GAP (World Health Organization, 2016). Each time a term was found, a score ‘1’ was assigned to the document. The maximum score possible for each country was 51.

#### Method 2: the governance framework of Anderson et al. (2019)

**Figure 1.**
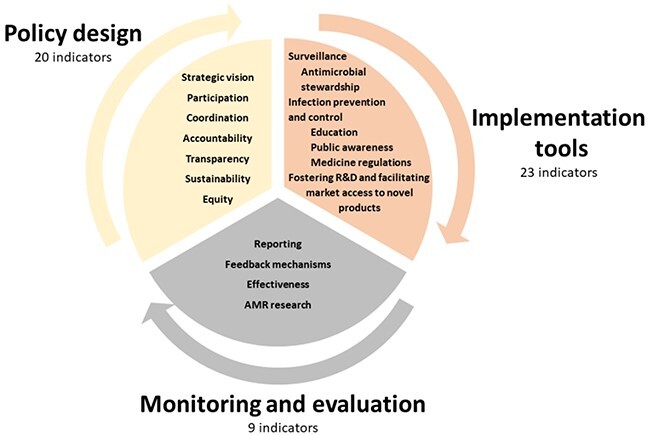
Schematic representation of the Governance framework proposed in Anderson *et al*. (2019), used for assessing the Latin American NAPs. The figure adapted with author permission from Anderson *et al.* (2019), shows the three governance areas of the framework (‘policy design’, ‘implementation tools’, and ‘monitoring and evaluation’) with their corresponding domains. The individual indicators can be found in Table 2. Arrows depict the cyclical feature of the framework

AMR governance in the NAPs was assessed following the framework in Anderson *et al*. (2019) consisting of three areas of governance: ‘policy design’, ‘implementation tools’ and ‘monitoring and evaluation’ (Figure 1). Each area is represented by domains and each domain can be assessed by indicators stated as questions, which ask for information about requirements for the domains. For example, transparency is a domain belonging to the ‘policy design’ area and the question ‘Is the complete NAP publicly available?’ is an indicator of the transparency domain (Table 2). Eighteen domains and 52 indicators form this governance framework (for a full description of governance areas, domains and indicators see Table 2). A complete justification of these indicators is given in Anderson *et al*. (2019).

Governance aspects addressed in the framework were quantified by assigning to each of the 52 indicators a score ‘1’ or ‘0’ indicating ‘presence’ or ‘absence’ of the requirements asked by each indicator, respectively. Since the aim of our scoring approach was to obtain an overall view of the aspects addressed in each NAP, we did not expect the NAPs to fulfil every requirement asked in every question stated as an indicator. A score of ‘1’ was assigned when the NAP addressed, either totally or partially, the information required by each indicator, and a score of ‘0’ was assigned when there was a total absence of the information asked (all scoring is reported in supplementary Table S2, see online supplementary material).

Since most NAP documents were divided into sections according to the strategic objectives defined in the GAP, the content search was addressed as a function of this to increase reliability in the findings. For example, when the content for the infection prevention and control (IPC) domain was evaluated, the focus was mainly, though not exclusively, on the activities described under the relevant strategic objective. Details of each NAP based on the activities of the indicators are given in supplementary Tables S3–S5, see online supplementary material.

#### Descriptive analysis of the NAPs

**Figure 2.**
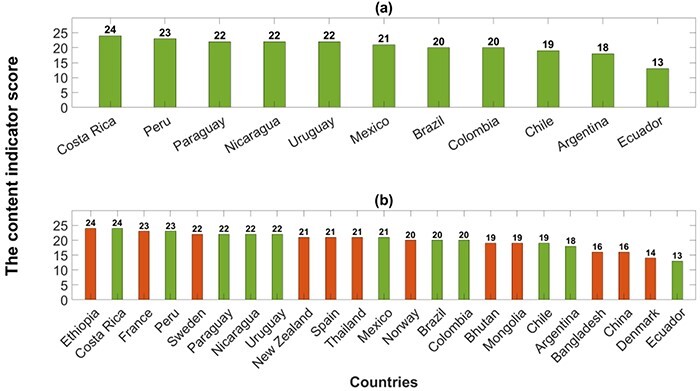
NAPs from Latin America lie within the group of NAPs least aligned with the GAP. Panel (a) shows the content indicator score of the Latin American countries under consideration (this study). Panel (b) includes (in orange) the countries with the lowest scores from the results obtained in Munkholm and Rubin (2020)

A descriptive analysis was carried out to summarise and visualize the scores obtained in the content analysis. The statistical methods used include frequency plots, measures of association for linear and monotonic correlations (Pearson’s and the Spearman’s rank correlation coefficients, respectively) and the mean (as a measure of central tendency). Scores for alignment of the NAPs with the GAP via the content indicator are presented in Figure 2a and situated within those obtained in Munkholm and Rubin (2020) for 59 NAPs published in English from the WHO regions: the African Region (AFRO), the South-East Asia Region (SEARO), the Western Pacific Region (WPRO), the Region of the Americas (PAHO), the Eastern Mediterranean Region (EMRO) and the European Region (EURO) (Figure 2b). The higher the score, the better the agreement of the NAP with the GAP. A NAP was arbitrarily considered to have a good alignment with the objectives of the GAP if at least 65% of the total possible score for the objectives (i.e. at least a score of eight out of the maximum score of 12) was achieved by the document. The same criterion was used for the corresponding actions. An association between the scores and the income level of the countries was also tested by the Spearman’s rank correlation coefficient (ρ) and the mean score of each income level was computed for comparison. Moreover, the mean score over the countries under consideration was calculated as indicative for comparison with other WHO regions.

**Figure 3.**
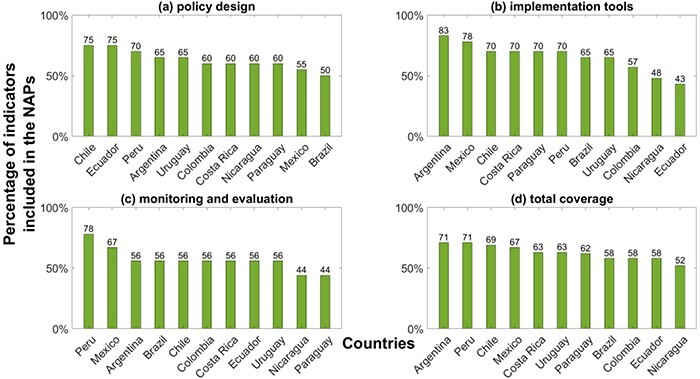
‘Monitoring and evaluation’ is the governance area least aligned with the framework of Anderson *et al.* (2019). Percentage of the framework indicators that are (totally or partially) covered by the NAPs. Panels (a-c) show results for each governance area separately, whereas panel (d) displays the overall coverage

A scoring approach to analyse the coverage of the Anderson *et al* (2019) framework indicators within the NAPs was also used. Similarly as above, an arbitrary cut-off threshold of 65% was defined. NAPs with at least 65% of the indicators with a score 1 were considered to have a good coverage. The coverage percentage was computed within each governance area as well as for the total of indicators (Figure 3). Additionally, Pearson’s correlation coefficient (*r*) was calculated to investigate whether there was a linear association between the score obtained from the two methods: the total score from the framework of Anderson *et al*. (2019) and the score of the actions from the content indicator in Munkholm and Rubin (2020). As the analysis conducted was descriptive rather than inferential statistical, no statistical significance was computed to compare differences between groups. Figures and calculations were done in MATLAB R2022a.

## Results

### Alignment of the NAPs with the GAP via the content indicator

Within the set of possible values for the content indicator (from 0 to 51), the score values obtained from the Latin American NAPs lie between 13 and 24, with Costa Rica ranked top and Ecuador with the lowest score (Figure 2a). Overall, the mean score was 20.4 and the standard deviation was 3. A negligible correlation was found between the score and the income level of the countries (ρ = −0.16). No significant differences were found for the mean score obtained per income level [22, 20.13 and 20.5 for lower-middle-income countries (LMICs), upper-middle-income countries (UMICs) and high-income countries (HICs), respectively] nor per geographic area (19.6, 23 and 21 for South America, Central America and Mexico, respectively). Note that most of the countries assessed are classified as UMICs by the World Bank (Table 1) and belong to South America. Also, whilst all WHO regions presented an overall mean score of 11.3 (out of a maximum of 12) for the objectives (see Methods section), no region exceeded a mean score of 18 (out of a maximum of 39) for the corresponding actions (Table 3).

**Table 3.** Mean score of the content indicator in Munkholm and Rubin (2020) for WHO regions shown for comparison

Region	Total	Objectives	Actions
AFRO	29.9	12	17.9
SEARO	29.4	11.8	17.5
WPRO	28	11.1	16.9
PAHO	27.7	11.3	16.3
EMRO	25.3	11.4	13.9
EURO	25.3	10.7	14.7
Latin America	20.4	11	9.4

Results for the African Region (AFRO), the Eastern Mediterranean Region (EMRO), the European Region (EURO), the Region of the Americas (PAHO), the South-East Asia Region (SEARO) and the Western Pacific Region (WPRO) were taken from Munkholm and Rubin (2020). Results for Latin America are from this study.

We then compared Latin American scores with the scores obtained from Munkholm and Rubin (2020) for other regions. NAPs from Latin America lie within the group of NAPs least aligned with the GAP, sharing a position with Ethiopia—ranked top together with Costa Rica—, then France, Sweden, New Zealand, Spain, Thailand, Norway, Bhutan, Mongolia, Bangladesh, China, and lastly Denmark with a score of 14 (Figure 2b). Figures of the scores by Munkholm and Rubin (2020) considering each WHO region separately are in supplementary Figure S1, see online supplementary material.

### Assessment of the NAPs via the governance framework

#### General description

The governance area ‘implementation tools’ is the most covered area across the Latin American NAPs, with eight countries covering totally or partially at least 65% of the indicators of this area (Figure 3b). The second most frequently addressed area is ‘policy design’, with five countries having at least 65% of the indicators of this area included in their NAPs (Figure 3a). The least reported governance area is ‘monitoring and evaluation’ (Figure 3c); only Peru and Mexico incorporated in their NAPs a significant number of indicators referring to this area (78 and 67% of the indicators, respectively), whereas Paraguay and Nicaragua addressed fewer than half of those indicators.

Argentina, Peru and Chile are always positioned in the top half of the list in Figure 3, i.e. within the NAPs covering more indicators of the governance framework in Anderson *et al*. (2019). This is reinforced when the total percentage of adhesion to the framework was observed (Figure 3d); in contrast, NAPs from Colombia, Ecuador and Nicaragua presented poorer alignment. Details of each NAP based on key activities of the indicators analysed are given in Tables S3–S5 for ‘policy design’, ‘implementation tools’ and ‘monitoring and evaluation’ governance areas, respectively.

#### Assessing aspects of governance

Based on the scoring approach, there were many governance aspects required by the framework that were addressed by all the Latin American NAPs analysed (Table S2). In the ‘policy design’ governance area, all NAPs incorporated the information comprised by coordination and participation domains to a greater or lesser extent (i.e. all aspects asked by the indicators belonging to these domains). Equity is also a domain well represented by all countries. In the accountability domain, all NAPs reported to a ministry or intersectoral committee accountable to the government for coordination and implementation. For the transparency domain, all NAPs are publicly available. On sustainability, all relevant sectors involved in each country committed to implementing the NAPs.

In the ‘implementation tools’ governance area, all countries considered the implementation of: a national surveillance system; stewardship programmes; IPC guidelines; educational programmes of AMR for relevant professionals; strategies for workforce capacity to deliver antimicrobial stewardship and IPC policies; public awareness campaigns; and regulations for appropriate antimicrobial use in animal health. Consistent with ‘policy design’ and ‘implementation tools’ areas, some components of the ‘monitoring and evaluation’ governance area were also covered by all countries, including indicators on reporting surveillance and NAPs progress, and on recognising research as a priority along with potential policies and interventions.

#### Gap analysis on aspects of governance

There were several requirements not mentioned in any Latin American NAP under consideration. From the documents, it is not clear whether there were agreements regarding the consequences of missing objectives set. There was no discussion of open access to the public for progress reports and funding allocations, and it was unclear whether this information would be in the public domain. There was no mention of a technical advisory group or subject matter experts for continuous advice during implementation and monitoring and evaluation stages. Incentives or penalties for IPC policies in human, animal and environment sectors were not mentioned. None of the documents mentioned dedicated budgets for research and development of novel antimicrobials and alternatives, diagnostic tools or vaccines. There was no reference to deadlines to review progress of particular actions and to discuss them across different levels.

#### The missing One Health approach

Our analysis showed that all Latin American countries indicated the need for strengthening and developing a national AMR surveillance system. However, only six countries (Costa Rica, Paraguay, Peru, Colombia, Nicaragua and Mexico) explicitly mentioned the environment sector within this context; whereas the human and animal sectors were unanimously considered (Table S2). With regard to the animal health sector, all countries outlined activities to regulate the use of antimicrobials in animals. However, only Costa Rica, Nicaragua and Paraguay specified actions for discouraging the marketing and against the use of antimicrobials as growth promoters, and only Uruguay has prohibited this use in animals (Government of Uruguay, 2017).

## Discussion

### Assessment of the content and governance framework of the Latin American NAPs

Overall, the strategic objectives of the Latin American NAPs were well aligned with the strategic objectives of the GAP, i.e. the vast majority of the key terms relative to the objectives of the GAP and NAPs overlapped. In contrast, poor alignment was observed between the corresponding actions in the NAPs and those recommended in the GAP; key terms relative to the actions in the GAP were less frequent in the NAPs. The governance aspects that were best addressed by the NAPs were the coordination, participation, equity and education domains. The domains that need to be developed further in subsequent NAPs included effectiveness, transparency, fostering research and development (R&D), and facilitating market access to novel products.

The discrepancy in alignment between the strategic objectives and the corresponding actions of the NAPs with respect to the GAP was not only observed for Latin America but also in other regions (Table S3). This discrepancy can be indicative of isomorphic mimicry, where government procedures appear to adopt best practices, but these practices are not implemented as intended (Munkholm and Rubin, 2020). The lack of alignment of NAPs with the actions of the GAP may be due to limitations of the keyword or key-phrase search done to identify relevant actions. The keywords and key-phrases used were chosen from the framework for action on AMR presented in the GAP. Whilst this framework necessarily establishes generic actions in correspondence with each strategic objective, the actions stated in the NAPs are not naturally generic, but describe the activities planned by the countries. The lack of precision of search terms in identifying relevant content has been reported as a limitation in content analysis (Lacy *et al*., 2015). In any case, alignment of the NAPs with the GAP matters as global coordination is needed to tackle AMR.

If we compare the results from Latin American NAPs with those from Southeast Asian NAPs (Chua *et al*., 2021) we can see that both regions share aspects that need to be strengthened, namely accountability, sustainability plans and transparency, international collaboration, and integration of the environmental sector. Within the accountability domain, we observed that in Latin America none of the countries mentioned what happens if objectives are not met. In the transparency domain, none of the countries mentioned public access to information on funding allocations, and only Peru, Ecuador and Chile mentioned a budget for determined activities (sustainability). Regarding international collaboration, 7 out of the 11 countries (Argentina, Costa Rica, Peru, Brazil, Ecuador, Uruguay and Mexico) alluded to the importance of being part of international surveillance systems for data sharing and collaboration (reporting domain). Nonetheless, since 1996 Latin America has had its own surveillance network, the Latin American Network for Antimicrobial Resistance Surveillance (ReLAVRA). The network manages AMR data for pathogens acquired in the community and hospitals with the purpose to inform AMR policies and IPC actions in Latin America. Surveillance data from food, animal and environment sectors are not included in the network database. ReLAVRA works together with the Global Antimicrobial Resistance and Use Surveillance System (GLASS).

### The One Health approach

The exclusion of the natural environment from AMR NAPs observed in this study—and specifically for rural Latin America (Medina-Pizzari *et al*., 2021)—is not an aspect relevant to Latin America only, but has been previously reported as a global issue (Iossa and White, 2018). Surprisingly, although the importance of the environment sector is highly emphasised in the Forward and Introduction sections of the GAP, there is no ‘Member State action’ (corresponding to the objectives of the GAP) that mentions the term environment. In the framework of Anderson *et al*. (2019), the inclusion of the environment sector is explicitly assessed by four indicators only (out of 52; Table 2) belonging to surveillance and IPC domains. The framework of Anderson *et al*. (2019) highlights the ban on antimicrobial use as an animal growth promoter to preserve the use of available antimicrobials in the EU. However, the situation in Latin America is different; from the NAPs analysed, only three countries (Costa Rica, Nicaragua and Paraguay) specified actions against this use, and only Uruguay has prohibited this use in animals (Government of Uruguay, 2017). AMR is not just a threat to human health but also to animal health and to the environment. The use of ineffective antimicrobials to treat sick animals has a negative impact on animal health and welfare (Bengtsson and Greko, 2014). Also, resistance can be transferred to the environment via human, animal and manufacturing waste (Hutchings *et al*., 2019), e.g. through the use of animal slurries as fertilisers. The environment can act as a reservoir of resistance, which inversely can be transferred to humans and animals (Fletcher, 2015). Further, animal production is an important source of transmission to humans via direct contact with animals and the food chain (World Health Organization, 2015a; Pokharel *et al*., 2020). In animal production, the consumption of antimicrobials is especially high—which favours resistance—as they are used not just for infection treatment but also prophylactically, and even as a growth promoter in some countries. All this emphasises the importance of taking a ‘One Health’ approach to tackle AMR (Pokharel *et al*., 2020).

### Methodological aspects of the analysis

Comparing AMR governance in Latin America with other regions is not trivial. Although other works have assessed NAPs via the framework of Anderson *et al*. (2019), their approaches differ considerably from our work, making a comparison among results unfeasible (Özçelik *et al*., 2022). For example, one study assessed the three governance areas of the framework, yet encompassed the framework indicators for the analysis of ‘implementation tools’ area only (Özçelik *et al*., 2022). In contrast, we fully used the framework to analyse the AMR strategy of the countries. In another study using the framework, the analysis was primarily based on the TrACSS data, assessing 60% of the framework indicators through the survey, whereas 32% of the indicators were evaluated through the NAP documents (Patel *et al*., 2023). In addition, unlike other studies, we manually translated the NAP documents. Language subtleties can be lost in machine translation, which is prone to word-sense errors (using the incorrect word for a given context, Kirchhoff *et al*., 2012). Additionally, some of us (PA, SAG, FL and MEFM) have cultural knowledge of the Latin American context, which is important in public health research (Carey, 1993).

Whilst results attained by using the framework in Anderson *et al*. (2019) can be more accurate, informative and detailed than the results by the content indicator in Munkholm and Rubin (2020), executing this approach to assess NAPs is time consuming. We computed the correlation between the scores obtained from the framework and the scores obtained for the actions from the content indicator; these scores are positively correlated albeit weakly (*r* = 0.28), which supports the use of the content indicator by Munkholm and Rubin (2020) for assessing NAPs on AMR.

The NAPs under consideration do not clearly distinguish among prior, ongoing and future activities; moreover, most NAPs included only a brief situation analysis. Also, progress has been made since the documents were published and the present analysis may not include this. For example, in 2022, Chile published the last version of its NAP, which is not considered in this study. Nonetheless, by analysing the documented plans in Latin America, this study has provided a basis for assessing implementation against policy intentions (Kayesa and Shung-King, 2021) in the region, and has given insight into the policy features that need to be reinforced. This supports the importance of carrying out an analysis on the TrACSS data for investigating the implementation state of the NAPs, which may be of help to establish policy priorities for the subsequent NAPs.

## Recommendations

The present study is a systematic content analysis of 11 Latin American NAPs on AMR published between 2015 and 2021. This analysis highlighted specific needs in the Latin American context where efforts could be concentrated to improve future NAPs. Based on this analysis and as a general outcome, which left aside the structural heterogeneity and asymmetries among the countries of the region, the subsequent Latin American NAP documents could:

- state actions to take if the objectives of the NAPs are not met;
- specify deadlines to review progress on specific actions of the NAPs, and make progress reports publicly available;
- set budgets for promoting AMR research and the development of novel antimicrobials, alternative treatments, diagnostic tools and vaccines;
- evaluate cost-effectiveness aspects of the NAPs by comparing the economic value between AMR actions and their effects through a measure such as the incremental cost-effectiveness ratio, which can help in making funding decisions;
- identify local experts for assistance throughout the stages of implementation, monitoring and evaluation to ensure sustainability of the NAPs;
- incorporate interventions for the environment such as programmes for waste management to reduce exposure to resistant organisms, financial and non-financial incentives for IPC policies, and surveillance systems for resistant organisms

## Conclusion

Here we present an overview of the policies developed in the NAPs on AMR in Latin America, identifying strengths and weakness, commonalities with other regions, and also providing a One Health perspective. One key finding was that the strategic objectives of the Latin American NAPs are well aligned with those in the GAP, while the corresponding actions are not. Following a governance framework, we were able to recommend the inclusion of particular actions in the subsequent NAP documents, e.g. actions for unmet objectives and activities for the environment sector. We could also identify governance commonalities with other regions that need to be strengthed, namely, accountability, sustainability plans and transparency, international collaboration, and integration of the One Health approach. This analysis may act as an incentive for other countries that have not released their NAPs. Finally, a better integration of the governance areas highlighted here in the future Latin American NAPs will allow for a more comprehensive suite of policies to tackle AMR

## Supplementary Material

czad118_Supp

## Data Availability

The data underlying this article are available online from the WHO AMR library, and in the article online supplementary material.

## Abbreviations

AMR = antimicrobial resistance

GAP = global action plan

HIC = high-income country

IPC = infection prevention and control

LMIC = lower-middle-income country

NAP = national action plan

R&D = research and development

ReLAVRA = Latin American Network for Antimicrobial Resistance Surveillance

TrACSS = Tripartite AMR country self-assessment survey

UMIC = Upper-middle-income country

WHO = World Health Organization

## Reflexivity statement

The inclusivity within the authors’ group is manifested in different aspects such as nationality (Argentina, Chile, Italy, UK), gender (two males and five females), areas of expertise, including biological sciences, veterinary, statistics, and policy, with experienced researchers and an early career researcher working in academia and governmental institutions. The authors have been working together—in their respective affiliations from Argentina and the UK—on translating science into policy practice.
