# Supplementary material for: National action plans on antimicrobial resistance in Latin America: an analysis via a governance framework
Source: Health Policy Plan. 2024 Jan 5;39(2):188–97. doi: 10.1093/heapol/czad118 (PMC10883663; doi:10.1093/heapol/czad118)
Supplement: czad118_Supp [file czad118_supp.zip › suppl_data/Table S3_Supplementary material.docx]

**Table S3. ‘Policy design’ governance area.** Actions of each NAP for the domains of this area

| **Country** | **STRATEGIC VISION** | **COORDINATION** | **PARTICIPATION** | **ACCOUNTABILITY** | **TRANSPARENCY** | **SUSTAINABILITY** | **EQUITY** |
| --- | --- | --- | --- | --- | --- | --- | --- |
| Argentina | -Situation of AMR in Argentina described in the NAP. Organisms subject to surveillance are mentioned. No measures of prevalence are reported in the NAP. The SIREVA-Argentina and PROVSAG networks provide information on AMR organisms in Argentina.  -NAP’s objectives are specific but not measurable nor time-bound.  -There are no quantitative targets but indicators are mentioned for surveillance of AMU and access: Defined Daily Dosis (DDD) of antimicrobial per inhabitant; rate of antimicrobial use per 100 consultations; percentage of antimicrobial prescription over the total of prescriptions; percentage of patients with at least one antimicrobial prescription over the total of patients; level of adaptation of antimicrobial prescriptions to health recommendations; percentage of users who received pharmaceutical services over the total of prescriptions and/or patients; percentage of users who register adherence to the indicated treatment; percentage of antimicrobial users without professional prescription. | -The Secretariat of Promotion and Health Programmes of the Ministry of Health coordinated the development of the NAP and different entities across sectors participated in the design (ANLIS, ANMAT, REMEDIAR, SENASA, INTA, Universidad Nacional de La Plata, Universidad del Centro de la Provincia de Buenos Aires, OIE, SADI, SATI).  -The National Commission for the Control of Antimicrobial Resistance of the Ministry of Health is responsible for verifying compliance with the NAP. Members: The National Directorate for Prevention of Diseases and Risks; ANMAT; INEI; ANLIS; INE; ANLIS; INTA; SENASA; El REMEDIAR + Redes; and The Directorate for Health Economy. Co-opted members: ADECI; SADI; SATI; OIE; OPS.  -Responsible entities were assigned for the activities of the plan. | -The topics of Surveillance, Regulation and Responsible use of AM, Prevention and control of infections were considered for human and animal health, and agri-food production sectors.  -Situation analysis, strategic lines and activities were defined for human and animal health, and agri-food production sectors.  -One Health approach mentioned as a model for the NAP. | - No mention of agreements regarding what happens if objectives are not met. | - Public access to AMR reports regarding human health available on the website [www.antimicrobianos.com.ar](http://www.antimicrobianos.com.ar) (data from the ReLAVRA, WHONET, SIREVA). | - No mention of dedicated budgets for the activities planned in the NAP.  - Providing supplies for the National Programme for Quality Control in Bacteriology and for the development of new diagnostic methodologies for multiresistant germs with specific budget allocation are activities mentioned in the NAP. | -Surveillance of access to antimicrobials to compare trends and differences in the rate of antibiotic use between regions and hospitals.  -Training and promotion of the rational and prudent use of antimicrobials for health professionals in outpatient and hospital settings.  -Dissemination in the community of the importance of the use of antimicrobials with medical prescription and adherence to the indicated treatment.  -Development of knowledge, training and information for veterinary professionals on the prudent and responsible use of antibiotics, based on pharmacokinetic, pharmacodynamics and toxicological bases. |
| Costa Rica | -Situation of AMR in Costa Rica described in the NAP. AMR organisms detected in hospitals, the community, livestock sector and pet animals are mentioned in the NAP. No measures of prevalence are reported in the NAP.  -NAP’s objectives are specific but not measurable nor time-bound.  -There are no quantitative targets nor indicators. | -The Ministry of Health coordinated the development of the NAP.  -The National Commission for the Fight against Antimicrobial Resistance of the Ministry of Health comprises entities across public and private sectors involved in AMU, and was responsible for the design of the NAP. | - One Health approach mentioned as a model for the NAP  -AMR to be included in training programmes within the One Health concept for relevant professionals.  -Regulations for surveillance, and prevention and control of the AMR by considering the One Health concept.  -Promote a common strategy for research within the One Health approach.  -Hygiene, prevention and infection control, including the One Health approach, to be incorporated as mandatory subjects in training programmes as well in the curricula of relevant professionals. | - No mention of agreements regarding what happens if objectives are not met. | - No mention of public access to progress reports, funding information, and AMR and AMU surveillance data.  - Some results for AMU in Costa Rica given in the NAP. | -No mention of dedicated budgets for the activities planned in the NAP.  - Designing a plan to ensure financial budget to implement the NAP is mentioned as an activity in the NAP. | -No mention of facilitating equitable access to existing essential antimicrobials.  -Ensure selection and access to new antimicrobials according to local epidemiology and criteria of financial, pharmacological and clinical sustainability.  -Responsible AMU incentivized by regulations including clinical guidelines for human health, animal and vegetal sectors; monitoring AM consumption; controlling prophylactic use to discourage their use as growth promoter. |
| Paraguay | -Situation of AMR in Paraguay described in the NAP. Resistance rates of organisms to determined antimicrobials are reported in the NAP.  -Detection of AMR organisms is established in Paraguay through the Central Laboratory of Public Health.  -NAP’s objectives are specific but not measurable nor time-bound.  -There are no quantitative targets nor indicators. | -The intersectoral Technical Committee for the Preparation of the National Antimicrobial Resistance Plan was responsible for the design.  -The committee is coordinated by the General Directorate of Health Surveillance of the Ministry of Public Health and Social Welfare, who are also responsible for the implementation of the Plan. | -One Health approach mentioned as a model for the NAP. | - No mention of agreements regarding what happens if objectives are not met. | - No mention of public access to progress reports, funding information, and AMR and AMU surveillance data. | -No mention of dedicated budgets for the activities planned in the NAP.  - Ensuring a financial budget for AMR surveillance via advocacy, and obtaining financing to implement the strategy by including the activities of the NAP within the governmental annual budget are activities mentioned in the NAP. | -No mention of facilitating equitable access to existing essential antimicrobials.  -Responsible AMU incentivized by regulations of post-marketing, dose and presentation, and by inclusion of educational programmes of appropriate AMU for professionals in human health, pharmaceutical industry to promote appropriate use, and other activities.  -Implement a traceability system in human and animal health to ensure the quality of the antimicrobials. |
| Peru | -Situation of AMR in Peru described in the NAP. Resistance rates of organisms to several antimicrobials are reported in the NAP. Incidence of some AMR organisms is compared to other regions.  -NAP’s objectives are specific, measurable and time-bound.  -Clear indicators for each activity mentioned and quantitative targets for some indicators defined. | -The National Group of Multisectoral Coordination is responsible for supervising, monitoring and assessment of the activities described in the plan.  -Responsible entities across human, animal and environment sectors were assigned for each activity of the plan. | -One Health approach mentioned as a model for the NAP.  -Design training modules for Health Sciences programmes and related subjects including the One Health approach. | - No mention of agreements regarding what happens if objectives are not met. | - No mention of public access to progress reports, funding information, and AMR and AMU surveillance data.  - Some results for AMR surveillance in Peru given in the NAP. | -There are dedicated budgets in place to implement specific activities in the NAP.  -Identify and distribute governmental and nor governmental funding sources to increase investment in new drugs, diagnostics, vaccines, surveillance and other interventions in the prevention and control of AMR are activities mentioned in the NAP. | -Promote rational use of antimicrobials by strengthening educational and training programmes, creating guidelines for clinical practices and pharmacological norms for use in humans.  -Strengthen regulations on AMU in animals, agriculture, aquaculture and food production sectors, and monitoring them.  -Strengthen good practices on appropriate AMU in hospitals. |
| Brazil | -No situation analysis described in the NAP.  -NAP’s objectives are specific but not measurable nor time-bound.  -There are no quantitative targets nor indicators. | -The Ministry of Health with the participation of multi-sectoral entities (Anvisa, MMA, Mapa, Funasa, MCTIC, MCidades, Opas/Brasil, CNS) participated in the design of the NAP.  -Responsible entities were assigned for each activity of the plan. | -One health approach mentioned as a model for the NAP.  -Promote strategies of communication and education for health professionals. | - No mention of agreements regarding what happens if objectives are not met. | - No mention of public access to progress reports, funding information, and AMR and AMU surveillance data. | -No mention of dedicated budgets nor assessment of future budget requirements to implement the activities in the NAP. | -Promote rational use of antimicrobials in human and animal sectors.  -Promote access to antimicrobials, vaccines and diagnostic tests in human health sector (tuberculosis is mentioned). |
| Colombia | -Situation of AMR in Colombia described in the NAP. Resistance rates of organisms to determined antimicrobials are reported in the NAP.  -NAP’s objectives are specific but not measurable nor time-bound.  -Indicators for each strategic objective stated. There are not quantitative targets. | -The Ministry of Health and Social Protection was in charge of the design of the plan with the actively participation of the Ministry of Agriculture and Rural Development, and the collaboration of different entities across sectors (ICA; Corcoipa; INS; INVIMA; OPS; ANDI).  -The Governance Board is responsible for implementation, monitoring and assessment of the Plan.  -Responsible entities were assigned for each activity of the plan. | -Human and animal health, phytosanitary control, and the environment sectors are mentioned as relevant actors for the NAP development. | - No mention of agreements regarding what happens if objectives are not met. | - No mention of public access to progress reports, funding information, and AMR and AMU surveillance data.  - Some results for AMR surveillance in Colombia given in the NAP. | -No mention of dedicated budgets for specific activities of the plan.  -Establishing financial requirements and budgets for the activities of the NAP is mentioned. | -Guarantee access to antimicrobials for human, animal health, and phytosanitary control by promoting quality and combating counterfeiting and/or smuggling.  -Design guidelines of antimicrobial in line with the guidelines of OIE, CIPF, OMS. |
| Ecuador | -Situation of AMR in Ecuador described in the NAP. AMR organisms are mentioned in the NAP. No measures of prevalence are reported in the NAP.  -NAP’s objectives are specific, measurable and time-bound.  -Clear indicators and quantitative targets for each activity defined. | -The Ministry of Health (as the coordinator) and different entities across sectors participated in the design of the NAP.  -Responsible entities were assigned for each activity of the plan. | -One Health approach mentioned as a model for the NAP  -Develop laboratory capacity for surveillance data | - No mention of agreements regarding what happens if objectives are not met. | - No mention of public access to progress reports, funding information, and AMR and AMU surveillance data. | -Dedicated budgets for specific activities of the plan to be defined. | -Ensure antimicrobial access of high quality in human and animal health sectors.  -Implement a sale traceability system in the animal health sector to ensure appropriate AMU.  -Training for pharmacy industry to ensure antimicrobials sales with prescription only. |
| Nicaragua | -Situation of AMR in Nicaragua described in the NAP. Resistance rates of organisms to various antimicrobials are reported in the NAP.  -NAP’s objectives are specific but not measurable nor time-bound.  -There are no quantitative targets nor indicators. | -The Ministry of Health (as the coordinator) with the participation of different institutions across sectors (INSS, IPSA, MAG, MARENA, ENACAL, MIFIC, public Universities) designed the NAP.  -Relevant authorities are responsible for the implementation of the NAP. | -One Health approach mentioned as a model for the NAP | - No mention of agreements regarding what happens if objectives are not met. | - No mention of public access to progress reports, funding information, and AMR and AMU surveillance data.  - Some results for AMR surveillance in Nicaragua given in the NAP. | -No mention of dedicated budgets for specific activities of the plan.  - Identify funding sources to implement the plan. | -Create mechanisms that guarantee access to antimicrobials of quality.  -Strengthen regulations on AMU in food production for animal consumption such as prophylactic use, and antimicrobial prescription.  -Discourage the use of antimicrobials as growth promoters. |
| Uruguay | -Situation of AMR in Uruguay described in the NAP. Resistance rates of organisms to various antimicrobials are reported in the NAP.  -NAP’s objectives are specific but not measurable nor time-bound.  -There are no quantitative targets nor indicators. An additional document will be developed to define indicators. | -The Technical Group for Antimicrobial Resistance Prevention of the General Directorate of Health (Ministry of Health) is responsible for the design and implementation of the NAP.  -Responsible entities were assigned for each activity.  -The Ministry of Livestock, Agriculture and Fisheries with the collaboration of institutions from the animal sector for interventions in animal health and food production chain.  -Additional documents with indicators for monitoring and assessment of the NAP. | -One Health approach mentioned as a model for the NAP | - No mention of agreements regarding what happens if objectives are not met. | - The Department of Laboratories of Public Health (DLSP) conducts surveillance of antibiotic resistance and collaborates with PulseNet. The data are send to ReLAVRA, and SIREVA.  - Some results for AMR surveillance in Uruguay given in the NAP. | -No mention of dedicated budgets for the activities of the plan.  -Determine budgets for distributing information about AMR and AMU through multimedia communication is mentioned for animal health. | -Monitoring prescription of antimicrobials.  -Promote the rational use by monitoring sales, and implementing programmes of optimal use in hospitals. |
| Mexico | -Situation of AMR in Mexico described in the NAP. There is no specification of resistance rates of organisms to antimicrobials in the NAP.  -NAP’s objectives are specific but not measurable nor time-bound.  -Quantitative targets stated (e.g. public awareness increase by 20%, AMU decrease in humans and animals by 20%) but there are no defined indicators. | -Intersecretarial Group on Antimicrobial Resistance (GIRAM) in charge.  -The General Health Council and the Scientific Advisory Commission of the General Health Council coordinated the elaboration of the plan.  -The intersciplinary group Federal Commission for the Protection against Sanitary Risks participated in the design. | -One Health approach mentioned as a model for the NAP | - No mention of agreements regarding what happens if objectives are not met. | - No mention of public access to progress reports, funding information, and AMR and AMU surveillance data. | -No mention of dedicated budgets in place to implement specific activities in the NAP.  -Assessment of the budget used by institutions for the activities of the strategy to be conducted.  -Showing the benefits of the strategy to strengthen budget by including national and international institutions in the implementation of the plan. | -Identify operational research priorities for responsible use.  -Promote rational use of antimicrobials by developing regulations and new policies for human and animal sectors. |
| Chile | -Situation of AMR in Chile described in the NAP. Organisms subject to surveillance are mentioned. No measures of prevalence are reported in the NAP.  -NAP’s objectives are specific, measurable and time-bound.  Clear indicators and quantitative targets for each activity defined. | -The Division of Disease Prevention of the Ministry of Health in charge for coordination.  -Different entities across sectors were responsible of the design of the NAP (Ministry of health, Ministry of Agriculture, Economy, Development and Tourism, scientific societies and universities). | -One Health approach mentioned as a model for the NAP  -Development of guidelines for IPC actions | - No mention of agreements regarding what happens if objectives are not met. | - No mention of public access to progress reports, funding information, and AMR and AMU surveillance data.  - Some results for AMR surveillance in Chile given in the NAP. | -Dedicated budgets in place for the implementation of a few activities. Budgets for most activities to be specified. | -Promote policies encouraging the responsible use of antimicrobials.  -Introduce new regulations and guidelines in human, animal and vegetal sectors.  -Identify economic incentives encouraging inappropriate use of antimicrobials in human and animal sectors. |
