# Supplementary material for: National action plans on antimicrobial resistance in Latin America: an analysis via a governance framework
Source: Health Policy Plan. 2024 Jan 5;39(2):188–97. doi: 10.1093/heapol/czad118 (PMC10883663; doi:10.1093/heapol/czad118)
Supplement: czad118_Supp [file czad118_supp.zip › suppl_data/Table S4_Supplementary material.docx]

**Table S4. ‘Implementation tools’ governance area.** Actions of each NAP for the domains of this area

| **Country** | **SURVEILLANCE** | **ANTIMICROBIAL STEWARDSHIP** | **INFECTION PREVENTION AND CONTROL** | **EDUCATION** | **PUBLIC AWARENESS** | **MEDICINES REGULATION** | **Fostering R&D and Facilitating Market Access to Novel Products** |
| --- | --- | --- | --- | --- | --- | --- | --- |
| Argentina | -Strengthening and continuing training for coordinating laboratories of the WHONET, SIREVA, and PROVSAG networks and laboratories participating in AMR surveillance.  -Development and implementation of the national AMR surveillance programme in food animals to determine the presence of resistance to different antimicrobials in at least two commensal bacteria: Escherichia coli and Enterococcus spp, and two zoonotic bacteria: Campylobacter spp and Salmonella spp.  -Harmonization of AMR surveillance strategies in human, animal, and environmental health.  -Design and implementation of surveillance of access to antimicrobials and appropriate use.  -Handbook for internal quality control of laboratories and programmes for external control. | -Online course on responsible AMU for medical doctors, nurses, pharmacists, dentists, biochemists and advanced students, delivered jointly by SADI, SATI, ADECI and the VIHDA programme, using the INE-ANLIS distance learning platform.  -Regional workshops on responsible AMU to disseminate surveillance, guidelines and treatment recommendations. Locations: Resistencia, Tucumán, Córdoba, Rosario, Mendoza, Bariloche, Puerto Madryn and Ushuaia.  -Prepare a good practice guide for the prescription and use of antimicrobials and other veterinary products, in line with international references in the animal sector.  -Organize training courses and workshops in collaboration with Universities, professional associations and technical exchange groups, for veterinarians and those who work in marketing chain, livestock farms and balanced feed production plants.  -Promote development and implementation of new rapid diagnostic methods for early detection of microorganisms with multiple resistance.  -Create laboratories with diagnostic protocols and networking to determine the level of resistance of bacteria isolated from food animals and the emergence of resistance mechanisms to critical antimicrobials in human and animal health, as well as the proper use of antimicrobials.  -Implement the National Day for the Responsible Use of Antibiotics to carry out actions promoting the recognition of the problem and its prevention. | -An IPC national programme in human health is established (The National Epidemiology Programme and Hospital Infection Control). It works on areas of education, research, regulations and surveillance.  -Practices on IPC to be strengthened, including activities of The National Hospital Infection Surveillance Program of Argentina (VIHDA).  -Regulations to implement infection prevention and control programmes between the Ministry of Agriculture, Livestock and Fisheries, the Ministry of Health, universities and professional associations to be developed.  -Training programmes and norms for biosecurity, hygiene and disinfection of personnel to be strengthened; creation of laboratories for bacterial surveillance in food animal.  -No mention of immunisation programmes.  -No mention of financial and non-financial incentives or penalties for IPC policies. | -The IPC national programme The National Epidemiology Programme and Hospital Infection Control includes areas of education, research, regulations and surveillance.  -Implement a distance learning course of responsible use of antimicrobials in human health to primary healthcare providers on the Argentina’s National Administration of Laboratories and Institutes of Health (ANLIS) platform, along with regional workshops about AMU, surveillance data, guidelines and recommended treatments.  -Include AMR in higher education curriculum and promote collaborative work between scientific community and healthcare professionals to design courses of prevalent infections and include the AMR topic in conferences.  -Implement a distance learning course of healthcare-associated infections on Argentina’s National Administration of Laboratories and Institutes of Health (ANLIS).  -Organise training courses and workshop of responsible use of antimicrobials for vets working in veterinary hospitals and clinics as well as in the food sector.  -Training on IPC for workforce in intensive animal production. | -Implement the National Day of the Responsible Use of Antibiotics to promote the recognition of the problem and its prevention.  -Create materials for dissemination (brochures, posters, articles, etc.) and campaigns on responsible use of antibiotics in the media.  -No mention of aspects of behavioural sciences, social science, and psychology. | -Implementation of measures that promote the proper use of antimicrobials according to scientific-technical precepts.  -Training for compliance with established standards on antimicrobial sales under filed prescription.  -Development or updating of regulations on the use of antimicrobials for preserving sensitivity bacteria as a non-renewable resource.  -The National Administration of Drugs, Foods and Medical Devices (ANMAT) agency is responsible for the regulation of the use of antimicrobials in human health, and The National Service for Agrifood Health and Quality (SENASA) in the animal health sector. | -Participation in the international conferences of the European and American Societies for Microbiology, and in other national and international scientific events.  -Participation in international discussion forums on regulatory practices for animal health.  -Prepare a good practice guide for the prescription and use of antimicrobials and other veterinary products, in line with international references.  -The INTA will contribute to research and development of alternative active ingredients to antibiotics such as growth factors and therapeutic agents in animal health and agri-food production sectors.  -Promotion of the development and implementation of new rapid diagnostic methods for the early detection of microorganisms with multiple resistance in human health.  -Development of a preferential evaluation procedure for new antimicrobials or new formulations and rapid diagnostic methods for resistant infections in human health.  -Access surveillance to antimicrobials through data on availability and use of drugs at outpatient and hospital levels, and on the commercialization of antimicrobials in the network of community pharmacies.  -No mention of dedicated budget for research and development of novel products. |
| Costa Rica | -Establish a National AMR surveillance programme for coordination, data collection and analysis.  -Introduce regulations for surveillance considering the One Health approach and organise surveillance for human health, animal, vegetable and environment sectors.  -Require surveillance of antimicrobial consumption, use and commercialization across human, animal, and vegetable sectors.  -Strengthen the national laboratory INCIENSA and develop laboratories of the Ministry of Agriculture and Livestock for AMR surveillance in animals and vegetables.  -No mention of capability supported by regular external quality assessments. | -Implement programmes to promote the prudent use of antimicrobials in human, animal, and vegetable areas.  -Promote that antimicrobial treatments are based on microbiological diagnostics and susceptibility tests.  -Support different developing national projects on the consumption and use of antimicrobials, and monitoring networks and surveillance.  -No mention of rapid diagnostic tools widely available and in regularly use. | -Establish a national IPC programme in human health, animal and vegetable sectors.  -Design policies and national regulations for decision-making in IPC.  -Promote research for IPC.  -Strengthen training programmes of hygiene and IPC for health care providers in human and animal health, and food handlers in food processing plants and vegetables sectors.  -Campaigns to promote hand-hygiene practices and to educate public for best practices to diminish spread of diseases.  -Strengthen supervision and control programmes in sewage water to improving environment health.  -Standardise methodology for final disposition of antimicrobials residuals.  -No mention of immunisation programmes.  -No mention of financial and non-financial incentives or penalties for IPC policies. | -Include AMR in higher education curriculum of human, animal and vegetable health programmes.  -Include the AMR and related topics in continuing training programmes for relevant professionals under the One Health approach.  -Include hygiene and IPC of infections under the concept One Health in training programmes for healthcare professionals and assistants, and in training programmes for food processing plants personnel. | -Disseminate AMR information through the media and social media networks and websites of relevant institutions.  -Design and develop campaigns on rational use of antibiotics for the general population.  -Establish a permanent programme of public communication and intensify the key messages in the World Antimicrobial Awareness Week.  -Estimate levels of awareness and knowledge on AMR through KAP-type studies (knowledge, attitude and practices) in different social and professional groups. | -Design regulations for the optimal use of antimicrobials, including guidelines for clinical practice to the most common infectious diseases.  -Design and implement electronic prescription and/or other control mechanisms for the prescription and use of antimicrobials in human, animal and vegetable health.  -Monitoring the antimicrobial consumption in human, animals and vegetables.    -Restrict the use of critical antimicrobials in human, animals and vegetables.  -Develop and update guidelines and protocols on the use of antimicrobials according to specific pathologies in human, animals and vegetables.  -Identify clinical situations where the prophylactic use of antimicrobials is required and design prophylaxis administration guidelines and protocols in human, animals and vegetables.  -Discourage the marketing and use of antimicrobials used as growth promoters in animals.  -No mention of an authority to monitor and enforce legislation having a dedicated budget. | -Research and development of new antimicrobials, alternatives and disease prevention methods.  -Ensure selection and access to current and new antimicrobials, according to local epidemiology, clinical criteria, pharmacological and financial sustainability.  -Promote that antimicrobial treatments are based on microbiological diagnostics and susceptibility tests.  -No mention of dedicated budget for research and development of novel products. |
| Paraguay | -Establish a National AMR surveillance programme and define relevant points in human, animal, agriculture and environment sectors.  -Workshops to discuss the scope of the national programme on detection of antimicrobial residues in raw and processed food, and detection of pathogenic bacteria and extraneous microbiota resistant.  -No specific mention of a national surveillance system for levels of antimicrobial use in animals and humans.  -Designate a National reference Laboratory for AMR surveillance in human, animals, agriculture and environment sectors, incorporating the state-of-the-art in equipment and technology with an external advisory board.  -Define specific pathogenic agents and resistance marker bacteria to be monitored in human, animal health sectors, environmental and food chain.  -Guarantee the quality of the laboratories supporting AMR surveillance through internal and external controls. | -Raise awareness and train on the use of antimicrobials in aquatic and terrestrial animal production and agricultural production.  -Prepare guidelines for the diagnosis, treatment and prevention of infectious diseases, including the prophylactic use of antimicrobials.  -Review, implementation and monitoring of guidelines for diagnosis and correct treatment of infections and appropriate use of antibiotics in disease management handbook (STIs, HIV, TB, Leprosy, Influenza, Malaria).  -Creation of public-private partnerships to promote research and development of diagnostic tests and other interventions.  -No mention of any use of financial and non-financial incentives or penalties in animal and human health to reduce inappropriate use of antibiotics. | -An infection control programme is established.  -Strengthen the programme of healthcare-associated infections.  -Dissemination of norms and protocols for the prevention and control of healthcare-associated infections.  -Continuously update prevention guidelines and regulations of healthcare-associated infections.  -Include modules of IPC, AMR prevention and patient safety in higher education for human health programmes, as well as modules of IPC in animal health and livestock production programmes.  -Ensure implementation of existing guidelines and regulations of IPC and prevention of AMR in human health, animal health and livestock production.  -Develop activities for awareness and dissemination of preventive actions to the public.  -Elaboration of regulations for disinfection and sterilization, hygiene and sanitation in hospitals.  -No mention of immunisation programmes.  -No mention of financial and non-financial incentives or penalties for IPC policies. | -Promote the inclusion of IPC modules in higher education curriculum for animal health and livestock production programmes.  -Development of training courses and specialities on IPC of infections and surveillance in animal health and livestock production for relevant professionals.  -Include AMR and related subjects in training courses for professionals in animal health, food industry and laboratory.  -Implement educational programmes on IPC of animal-associated infections for the community.  -Continuing training programmes for antimicrobial prescribers and dispensers and member if the network. | -Design, implementation and assessment of a communication campaign.  -Production of awareness material for healthcare professionals and workers.  -Introduction of new technological tools for dissemination of information.  -Establish a plan about rational use of antimicrobials to be incorporated into other strategies such as “Healthy Family”. Carry out workshops about the plan and deliver it via publications, media and social media networks.  -Implement a communication program on the rational use of antimicrobials in the livestock sector, and train producers on rational use of antimicrobials.  -Estimate levels of knowledge, attitude and practices on AMU in certain population to define actions for campaign. | -Supervise compliance with current regulations on the use of antimicrobials in the production of animals, vegetables, and processed foods.  -Verify application of regulations and laws in force for surveillance and quality control of antimicrobials.  -Create policies for appropriate use of antimicrobial agents in health care.  -Develop guidelines for the appropriate use of antimicrobial agents in human health care.  -Eliminate the use of antimicrobials (growth promoters) in animals intended for human consumption and in agriculture, and develop alternative products. | -Promote channelling of government resources and not governments to increase investment in new medicines, diagnostics, vaccines and other interventions in the prevention and control of AMR.  -Promote management strategies for access to vaccines at all levels, with emphasis on small-scale producers of animals for consumption.  -No mention of dedicated budget for research and development of novel products. |
| Peru | -Implement an integrated national AMR surveillance system and define roles and tasks for each sector involved: MINSA, EsSalud, MINAGRI, SENASA y SANIPES FFAA, FFPP, Professional colleges, Universities, Private institutions, SENASA y SANIPES, Regional and local governments.  -Determine microorganisms and antimicrobials to monitor in human, animal, environmental and food chain health.  -Implement a national system of laboratories for AMR surveillance in human health, animal health, food chain and environment.  -Organize a national programme to guarantee the quality of the diagnostics of the laboratories including strengthening of the external quality assessment programmes for diagnostic confirmation and detection of AMR. | -Promote the rational use of antimicrobials in patients of health establishments.  -Strengthen technological capacity through the innovation of diagnostic methods, high-tech equipment and HR training.  -Standardize laboratory diagnostic methods for the identification and characterization of the agents involved in AMR.  -Assess economic incentives that encourage inappropriate use of antimicrobials.  -No mention of any use of financial and non-financial incentives or penalties in animal and human health to reduce inappropriate use of antibiotics. | -Include modules of IPC and AMR in higher education for human and animal health professionals.  -Design a timeline for monitoring prevention and control of healthcare-associated infections, implement it and create reports.  -Strength IPC system in primary care services.  -Promote personal hygiene practices at different levels including the food chain.  -Updating, implementing and monitoring regulations on good hygiene practices throughout the food chain.  -No mention of immunisation programmes.  -No mention of financial and non-financial incentives or penalties for IPC policies. | -Promote training on AMR for healthcare professionals in higher education and continuing education programmes.  -Promote that healthcare science programmes include related topics on rational use of antimicrobial and reducing AMR.  -Include IPC of infections in higher education programmes and training courses.  -Implement continuing medical education programmes on rational use of antimicrobials for prescribers of healthcare services.  -Strengthen higher education curriculum for professionals in human health, animal health, agriculture and food industry about rational use of antimicrobials. | -Promote communications on control of AMR and rational use of antimicrobials in scientific conferences.  -Identify previous communication campaigns that have had a positive impact on reduction of AMR and explore the continuity of those for rational use of antimicrobials.  -Highlight topics addressed in campaigns via publications in the media.  -Establish a platform on information about antimicrobials for consumers.  -No mention of aspects of behavioural sciences, social science, and psychology. | -Supervise compliance with regulations on the use of antimicrobials in human, animal production, agriculture, aquaculture and processed foods.  -Develop and implement clinical practice guidelines for frequent infections at each stage of life, which promote the rational use of antimicrobials in humans.  -Establish pharmacological standards for health registration and rational use of antimicrobials in humans.  -Strengthen the specific sanitary regulations related to the use of antimicrobials in the production of food animals (terrestrial and aquatic) and agriculture.  -Strengthen actions against the illegal trade of antimicrobials intended for use in animals and agriculture. | -Promote channelling of government resources and not governments to increase investment in new medicines, diagnostics, vaccines and other interventions in the prevention and control of AMR.  -Prepare and execute projects integrated with the laboratory networks, health and production services, for the development of new methods for the detection of AMR and the validation of methods existing internationally.  -No mention of dedicated budget for research and development of novel products. |
| Brazil | -Create an integrated surveillance system of AMR.  -Define and implement a network of laboratories for surveillance of susceptibility of microorganisms and antimicrobial use in humans and animals.  -No mention of adequate laboratory capacity and capability supported by regular external quality assessments. | -Elaborate and implement national guidelines on rational use of antimicrobials.  -Encourage creation of committees for the rational use of medicines in counties.  -Evaluate diagnostic methods for timely identification of AMR in health services.  -No mention of any use of financial and non-financial incentives or penalties in animal and human health to reduce inappropriate use of antibiotics. | -Perform situation analysis for the current IPC strategies.  -IPC policy including objectives, strategies of implementation, monitoring and evaluation to be proposed.  -Promote the implementation of the national programme for the prevention and control of healthcare-associated infections (PNPCIRAS).  -Disseminate safe practices for infection prevention and support its implementation in health services.  -Maintain and expand the vaccination coverage by territory, age group and immunobiological targets agreed in the National Immunization Programme.  -Strengthen the adoption of good agricultural practices, including regulatory strategies.  -Strengthen public sanitation management, and the policy on basic sanitation, expand treatment of sanitary wastewater and access to drinking water, according to the National Basic Sanitation Plan (Plansab).  -No mention of financial and non-financial incentives or penalties for IPC policies. | -Promote and support continuous training on AMR for professionals and admin staff in human, animal, and environment health sectors.  -Include the AMR and related topics in higher education curriculum for professionals in human, animal and environment health. | -Include AMR theme in the curriculum of primary schools.  -Develop and implement National Communication Plan on the AMR theme for professionals and managers working in the areas of human, animal and environmental health, civil society, the regulated sector and social movements.  -Give guidelines periodically to different sectors on prevention and control of AMR.  -No mention of aspects of behavioural sciences, social science, and psychology. | -Strengthen strategies of the National Policy of Pharmaceutical Assistance that promote the rational use of medicines with a focus on the use of antimicrobials.  -Strengthen regulatory actions to promote the rational use of antimicrobials in animals. | -Induce scientific, technological and industrial development related to AMR products.  -Stimulate and promote the development, production and maintenance of the productive capacity in the pharmaceutical, chemical and biotechnological industry, production of medicines, diagnostic methods and vaccines.  -Strengthen public producers and infrastructure for the production and innovation of essential antimicrobials, with emphasis on the production of drugs with low economic profitability.  -No mention of dedicated budget for research and development of novel products. |
| Colombia | -Strengthen the AMR surveillance system by establishing a multisectoral technical group, including mechanisms for data analysis and ensuring national coverage of surveillance systems (resistance surveillance including sanitary, epidemiological and laboratory surveillance).  -Strengthen surveillance capacities in public health related to AMR (human and animal health, phytosanitary control and environment).  -Strengthen the capacity of the national reference laboratories for AMR and guarantee implementation of quality management systems, including external assessment. | -Develop programmes to optimize the use of antimicrobials in health care institutions.  -Perform a diagnostic of the regulatory framework corresponding to the prescription and sale of antimicrobials in the agricultural sector.  -Design strategies to promote the optimal use of antimicrobials in the agricultural sector.  -No mention of rapid diagnostic tools widely available and in regularly use.  -No mention of any use of financial and non-financial incentives or penalties in animal and human health to reduce inappropriate use of antibiotics. | -Create and implement a national IPC programme and IPC guidelines.  -Implement the Healthy Environments Strategy.  -IPC training for health care providers in human and animal health, livestock production and agriculture.  -Promote actions of sanitation, hygiene and disinfection in primary production, food production and environmental sanitation.  -Strengthen health and safety regulations in animal and vegetal production.  -Strengthen regulations of the processing and commercialization of food, medical devices, medicines and antimicrobials, water resources, and prevent environmental pollution.  -Strengthen programmes and guidelines of prevention and control of officially controlled diseases in animals.  -No mention of immunisation programmes.  -No mention of financial and non-financial incentives or penalties for IPC policies. | -Include IPC of infections and rational use of antimicrobials modules in higher education curriculum for human, animal health and phytosanitary control programmes.  -Design and implement strategic for continuous training of IPC of infections and AMR for health care workforce in human, animal, livestock and agricultural production sectors.  -Implement training of national regulation on antimicrobial prescription to the community. | -Establish a communication programme for general population to promote safe use and consumption of antimicrobials based on scientific evidence that includes relevant sectors.  -Include activities for IP and proper use of antimicrobials in primary and secondary schools.  -Strengthen the concepts of IPC of infections and rational use of antimicrobials in the curriculum of further and higher education for professionals of human and animal health and phytosanitary control.  -No mention of aspects of behavioural sciences, social science, and psychology. | -Carry out a diagnosis of the regulatory framework corresponding to the dispensing of antimicrobials to the community in general by dependent and independent pharmaceutical services.  -Manage strategic alliances with actors in the productive sector to promote compliance with the regulation of the use of antimicrobials in the agricultural sector. | -No mention of activities and budget for research and development of novel products. |
| Ecuador | -Strengthen the AMR surveillance system in human health and design a surveillance system in animal health.  -Develop the capacity of laboratory to produce high quality microbiological data for AMR surveillance with the One Health approach.  -Strengthen the national centre reference of AMR involving frequently update of microorganisms for surveillance.    -Designate a laboratory as a centre of reference for agricultural sector.  -No mention of adequate laboratory capacity and capability supported by regular external quality assessments. | -Design and disseminate strategies to promote the optimal use of antimicrobials.  -Prepare a plan of clinical practice guidelines for the most frequent pathologies for the use of antimicrobials.  -Register and control establishments selling products for veterinary use.  -Training on the proper use of veterinary drugs.  -No mention of rapid diagnostic tools widely available and in regularly use.  -No mention of any use of financial and non-financial incentives or penalties in animal and human health to reduce inappropriate use of antibiotics. | **-I**mplement a national IPC programme for health care sector.  -Implement multimodal hand hygiene strategy.  -Include IPC of infection in programmes for training and education of animal health professionals.  -No mention of immunisation programmes.  -No mention of financial and non-financial incentives or penalties for IPC policies. | -Include the AMR theme in higher education within the Responsible Autonomy Framework. | -Communication of diagnosis on AMR in a related population and relevant professionals in human and animal health, environment, agriculture, aquaculture and fishing sectors.  -Design a permanent agenda of communication on AMR topics.  -Participate in the World Antimicrobial Awareness Week.  -Development of radio programmes with identified spokespersons.  -No mention of aspects of behavioural sciences, social science, and psychology. | -Regulate and control antimicrobial prescription, dispensation and sale.  -Implement optimization programmes of antimicrobial use in hospitals.  -Strengthen the control of the production-consumption chain.  -Training on antimicrobial sales under mandatory prescriptions for pharmacy staff.  -Develop regulations for the use of critical antimicrobials in animals. | -No mention of activities and budget for research and development of novel products. |
| Nicaragua | -Form a multisectoral technical group to establish an integrated surveillance system of AMR.  -Develop and implement an integrated surveillance system of AMR for human, animal, vegetables, food and environment.  -Strengthen the capacities of laboratories to produce quality microbiological data for the management and activities of integrated surveillance in AMR.  -No mention of regular external quality assessments of adequate laboratory capacity and capability. | -Sensitize stakeholders on the ethical commerce of antimicrobials for agricultural use.  -Perform a diagnostic of the regulatory framework corresponding to production, good practices, storage and distribution, and use of antimicrobials.  -Identify critical antimicrobials for surveillance of their consumption and resistance, taking into account the lists of antimicrobials issued by the WHO and OIE.  -No mention of rapid diagnostic tools widely available and in regularly use.  -No mention of any use of financial and non-financial incentives or penalties in animal and human health to reduce inappropriate use of antibiotics. | -Establish a national IPC programme in human, animal and vegetable health, agroindustry and environment.  -Strength training programmes on biosafety, water, sanitation, hygiene, food, medicine, environment, prevention, disease and pest control.  -Review and update the regulations on human, animal and vegetable health, agroindustry and environment.  -Strengthen guidelines for the prescription and rational use of antimicrobials.    -Promote good practices in primary production, biosafety, food safety, agricultural and agro-industrial establishments.  -No mention of immunisation programmes.  -No mention of financial and non-financial incentives or penalties for IPC policies. | -Include IPC of infections and rational use of antimicrobials modules in higher education curriculum for professionals and technical stuff in human and animal health, vegetables, food, medicines and environment sectors.  -Develop continuous training programmes on AMR for students, professionals and technical staff.  -Include activities promoting care and self-care for the prevention of infections and proper use of antimicrobials in primary, secondary and Further Education.  -Implement the environmental education plan.  -Strengthen training programmes of biosecurity, water, sanitation, hygiene, food, medicine, environment, prevention, disease and pest control for professionals and technical staff. | -Establish a permanent communication program for the general population that promotes safe use and consumption of antimicrobials.  -Implement the Hand Hygiene strategy.  -Strengthen key messages of the National Action Plan for the Containment of Antimicrobial Resistance during the Word Hand Hygiene Day, Word Environment Day, Word Food Safety Day and World Antimicrobial Awareness Week.  -No mention of aspects of behavioural sciences, social science, and psychology. | -Carry out a diagnosis of the regulatory framework corresponding to the production, good practices, storage, dispensing, and use of antimicrobials.  -Update, adapt and develop legislation related to the use of antimicrobials used in the food production for animal consumption.  -Promote reduction of the use and commercialization of antimicrobials as growth promoters or performance enhancers, in all animal species for consumption.  -No mention of an authority to monitor and enforce legislation having a dedicated budget. | -No mention of activities and budget for research and development of novel products. |
| Uruguay | -Develop an integrated system of AMR surveillance including human and animal health sectors responsible for data collection and analysis.  -Identify resistant microorganisms of priority for the country.  -Strengthen the capacity of National Reference Laboratories and identify laboratories that can be included in the network.  -No specific mention of surveillance for levels of antimicrobial use in animals and humans.  -No mention of regular external quality assessments of adequate laboratory capacity and capability. | -Control the use of critical antibiotics and antimicrobial sales in the human sector.  -Promote the use of rapid, simple and inexpensive diagnostic tests to minimize the delay in initiation of treatment in animals.  -Creation of instances of participation with industry for the control of antimicrobial sales.  -Deliver courses and develop guidelines and protocols on the prevention of resistant infections, diagnostic of resistant organisms, implementation of good practices for antimicrobial use, and proper management of the control and spread of AMR in animals.  -Update protocols for laboratory diagnostic.  -No mention of any use of financial and non-financial incentives or penalties in animal and human health to reduce inappropriate use of antibiotics. | -Strengthen the committees of hospital IFC (CIH (Spanish)) through external audits.  -Training for health care workers and professionals and awareness of hand hygiene.  -Implementation of protocols for surgical prophylaxis.  -Strengthen the citizen vaccination campaign.  -Training for the primary care services, community survey of frequent infections, educational programmes on health-care associated infections.  -No mention of financial and non-financial incentives or penalties for IPC policies. | -Include activities on AMR in the curriculum for primary and secondary schools.  -Include AMR in higher and further education curriculums.  -Participate together with public and private agents continuous training programmes on AMR.  -Strengthen CIHs (Committees of IPC of hospital infections) through courses and workshops for continuous training. | -Strengthen the citizen vaccination campaign as a IPC measure.  -Include activities related to the AMR in curriculum of primary and secondary schools.  -Participate in World Antimicrobial Awareness Week.  -Spread information about AMR, rational use of antimicrobials, and health risks associated.  -No mention of aspects of behavioural sciences, social science, and psychology. | -Strengthen legislation/programs that promote the correct dispensing and use of antimicrobials. | - No mention of activities and budget for research and development of novel products. |
| Mexico | -Strengthen the multisectoral group for surveillance coordination.    -Strengthen programmes for epidemiological surveillance and surveillance of antimicrobial use in human health, animal health and environment.  -Establish catalogues of microorganisms of interest for epidemiological surveillance of AMR in hospitals, community and food settings, as well as in animals and in the environment.  -Establish surveillance systems for antimicrobial consumption in hospitals, community and animal sector.  -Designate reference laboratories for AMR surveillance in human, animal, and environmental health.  -No mention of regular external quality assessments of adequate laboratory capacity and capability. | -Integrate and strengthen in clinical guidelines the issue of rational use of antimicrobials in private and public institutions.  -Promote updating of the official Mexican standards for infectious diseases with a focus on rational use of antimicrobials for human and animal use.  -Boost the sanitary surveillance strategy regarding sales and dispensing of antibiotics for human and animal use.  -Include training on rational use of antimicrobials as criterion for certification and re-certifications of medical doctors.  -Inclusion of national and international research institutions in the implementation of the plan to support the development of new medicines, therapeutic alternatives, diagnostic tools, vaccines and other interventions.  -Promote antibiotic management programmes in all public and private hospitals. Document and exchange successful experiences in the implementation of these programmes.  -No mention of any use of financial and non-financial incentives or penalties in animal and human health to reduce inappropriate use of antibiotics. | -Promote vaccination in humans.  -Use zoosanitary measures and promote good practices in livestock, aquaculture and fishing production units.  -Strengthen coordination for prevention of zoonotic diseases.  -Promote good hygiene practices and food manufacturing for consumption human and animal.  -Integrate and implement a national programme for epidemiological surveillance, prevention and control of healthcare associated infections.  -Permanently strengthen hand hygiene in public and private health services.  -Coordinate a National Strategy for Basic Sanitation and Hygiene in the Community.  -No mention of financial and non-financial incentives or penalties for IPC policies. | -Promote the inclusion of the topics AMR and AMU in primary and secondary schools, and in higher education programmes for human and animal health professionals.  -Develop and deliver continuous training courses for human and animal health professionals.  -Include AMU training as criteria for the certification and re-certification of medical doctors.  -Training for operators of water supply systems to promote disinfection of water for human use and consumption. | -Develop a national campaign on AMU to be held every November within the framework of the World Antimicrobial Awareness Week, including informative spots in TV, radio and social media networks.  -Distribute information about AMR and AMU in Firstcare Practices (in particular for acute respiratory infections and acute enteric infections), and in waiting rooms of hospitals, especially during the World Antimicrobial Awareness Week.  -Develop an intersectoral website on AMR with content for the general population and other sectors.  -Promote the inclusion of AMR theme and rational use of antimicrobials in the curriculum of primary and secondary schools.  -Assess knowledge on AMR and knowledge, attitude and practices on AMU in different groups of the population. | -Establish a Consultative Committee of Experts for the development of policies on the rational use of antimicrobials.  -Update norms of infectious diseases with a focus on rational use of antimicrobials for human and animal use.  -Development of a national policy on rational use of antimicrobials in veterinary practice and animal production.  -Strengthen the regulation on the marketing and selection of antimicrobial products and diagnostic methods for infectious diseases.  -Promote regulatory policies for proper management of antimicrobial waste.  -No mention of an authority to monitor and enforce legislation having a dedicated budget. | -Inclusion of national and international research institutions in the implementation of the plan to support the development of new medicines, therapeutic alternatives, diagnostic tools, vaccines and other interventions.  -Exchange experiences and information with the pharmaceutical industry to promote the development of new medicines, diagnostic tools, vaccines and others interventions.  -No mention of dedicated budget for research and development of novel products. |
| Chile | -Establish a National AMR surveillance programme and generate information of AMR in human health.  -Develop laboratory capacity for surveillance in human and animal health sectors.  -Strengthen the National Reference Laboratory and the programme of external quality assessment. | -Update the national list of essential medicines.  -Promote policies that encourage the prudent and responsible use of antimicrobials based on intergovernmental standards and guidelines.  -Identify and analyse economic incentives that promote the inappropriate use of antimicrobials in human and animals.  -The department of transmissible diseases of the Division for Disease Prevention and Control (DIPRECE) is in charge of designing the Clinical Practice Guidelines for antimicrobial use of the most common diseases and has a dedicated budget. | -Strengthen the programme for health-care associated infections.  -Establish intergovernmental regulations and guidelines regarding IPC under the One Health approach.  -Strengthen programmes for control of infections, including AMU in animal product production.  -Laboratory support for proper AM prescription and timely infection detection.  -Establish strategies for programmes of education, training and development on IPC.  -Restrict development and dissemination of AMR outside healthcare environments by ensuring potable water supply, reduce sexually transmitted diseases and strength vaccination programmes.  -No mention of financial and non-financial incentives or penalties for IPC policies. | -Include AMR in higher and further education curriculums for human and animal health professionals.  -Design online courses for human health professionals.  -Develop training programmes for making good use of antimicrobials through institutions that support producers. | -Develop a public campaign in the media and other social events to promote the good use of antimicrobials in human and animals.  -Commemorate the Day against antimicrobial resistance.  -Include the AMR topic in scientific conferences and promulgate the antimicrobial use strategy within scientific communities.  -Develop educational programmes on AMU for pet owners.  -Disseminate and raise awareness about AMR in schools.  -Establish a coordination plan with pharmacies for them to act as agents of prevention and control of AMU.  -No mention of aspects of behavioural sciences, social science, and psychology. | -Monitoring antimicrobial use through implementation of electronic prescriptions, prevalence assessments and test of antimicrobials suitability.  -Regulate and control promotional practices in industry.  -Develop regulations for the use of antimicrobials in domestic animals.  -Ensure that the acquisition and prescription of antimicrobials is ruled and supported by regulations.  -Establish regulation on antimicrobial used for domestic animals. | -Strengthen existing public-private partnerships and create new ones to promote research and development of new antimicrobial agents, vaccines, and diagnostic methods.  -Participate in national and international collaborative activities for the investigation of natural sources of biodiversity and biological repositories as sources of new antimicrobial agents.  -No mention of dedicated budget for research and development of novel products. |
