# Supplementary material for: National action plans on antimicrobial resistance in Latin America: an analysis via a governance framework
Source: Health Policy Plan. 2024 Jan 5;39(2):188–97. doi: 10.1093/heapol/czad118 (PMC10883663; doi:10.1093/heapol/czad118)
Supplement: czad118_Supp [file czad118_supp.zip › suppl_data/Table S5_Supplementary material.docx]

**Table S5. ‘Monitoring and evaluation’ governance area.** Actions of each NAP for the domains of this area

| **Country** | **REPORTING** | **FEEDBACK MECHANISMS** | **EFFECTIVENESS** | **AMR RESEARCH** |
| --- | --- | --- | --- | --- |
| Argentina | -Annual progress on the NAP reported on WHO website via the TrACSS.  -Annual surveillance reports are sent to the PAHO and are published on the website [www.antimicrobianos.com.ar](http://www.antimicrobianos.com.ar) | - No mention of regular deadlines to review progress on activities and feedback. | - No mention of cost-effectiveness evaluation for policies and interventions implemented. | -Both AMR research and policies aspects are identified as key priorities.    -There is no dedicated national budget for AMR research. |
| Costa Rica | -Annual progress on the NAP reported on WHO website via the TrACSS.  -Participate in national and international projects to exchange information about AMR. | - No mention of regular deadlines to review progress on activities and feedback. | - No mention of cost-effectiveness evaluation for policies and interventions implemented. | -Both AMR research and policies aspects are identified as key priorities.    -There is no dedicated national budget for AMR research. |
| Paraguay | -Annual progress on the NAP reported on WHO website via the TrACSS. | - No mention of regular deadlines to review progress on activities and feedback. | - No mention of cost-effectiveness evaluation for policies and interventions implemented. | -Both AMR research and policies aspects are identified as key priorities.    -There is no dedicated national budget for AMR research. |
| Peru | -Annual progress on the NAP reported on WHO website via the TrACSS.  -Implementation of a research and technological innovation programme applied to the comprehensive surveillance of AMR including agreements with international institutions. | - No mention of regular deadlines to review progress on activities and feedback. | -Clear indicators and quantitative targets that could inform effectiveness.  -Analysis of cost-effectiveness, cost-utility or budgetary impact of the adoption of new AMR diagnostic methods in human, animal, and environmental health. | -Both AMR research and policies aspects are identified as key priorities.    -There is no dedicated national budget for AMR research. |
| Brazil | -Annual progress on the NAP reported on WHO website via the TrACSS.  -Develop management and interoperability between the information systems of the Ministry of Health with the national AMR monitoring system and the national AMR monitoring system with those International. | - No mention of regular deadlines to review progress on activities and feedback. | - No mention of cost-effectiveness evaluation for policies and interventions implemented. | -Both AMR research and policies aspects are identified as key priorities.    -There is no dedicated national budget for AMR research. |
| Colombia | -Annual progress on the NAP reported on WHO website via the TrACSS. | - No mention of regular deadlines to review progress on activities and feedback. | -Indicators of effectiveness defined for each strategic line.  -No mention of cost-effectiveness evaluation for policies and interventions implemented. | -Both AMR research and policies aspects are identified as key priorities.    -There is no dedicated national budget for AMR research. |
| Ecuador | -Annual progress on the NAP reported on WHO website via the TrACSS. | - No mention of regular deadlines to review progress on activities and feedback. | -Clear indicators and quantitative targets that could inform effectiveness.  -No mention of cost-effectiveness evaluation for policies and interventions implemented. | -Both AMR research and policies aspects are identified as key priorities.  -Develop an agenda for AMR research in health sectors, agriculture, environment and aquaculture to obtain information not found by surveillance.  -There is no dedicated national budget for AMR research. |
| Nicaragua | -Annual progress on the NAP reported on WHO website via the TrACSS. | - No mention of regular deadlines to review progress on activities and feedback. | - No mention of cost-effectiveness evaluation for policies and interventions implemented. | -Both AMR research and policies aspects are identified as key priorities.  -There is no dedicated national budget for AMR research. |
| Uruguay | -Annual progress on the NAP reported on WHO website via the TrACSS.  -The Department of Laboratories of Public Health (DLSP) conducts surveillance of antibiotic resistance and sends the surveillance data to ReLAVRA, and SIREVA. It also collaborates with PulseNet. | - No mention of regular deadlines to review progress on activities and feedback. | - No mention of cost-effectiveness evaluation for policies and interventions implemented. | -Both AMR research and policies aspects are identified as key priorities.  -There is no dedicated national budget for AMR research. |
| Mexico | -Annual progress on the NAP reported on WHO website via the TrACSS.  -Link the national AMR reference laboratories with the networks of reference centres of international organizations. | - No mention of regular deadlines to review progress on activities and feedback. | -Carry out continuous evaluations on the cost-effectiveness of the strategies defined against AMR. | -Both AMR research and policies aspects are identified as key priorities.  -There is no dedicated national budget for AMR research. |
| Chile | - Annual progress on the NAP reported on WHO website via the TrACSS. | - No mention of regular deadlines to review progress on activities and feedback. | -Clear indicators and quantitative targets that could inform effectiveness.  -No mention of cost-effectiveness evaluation for policies and interventions implemented. | -Both AMR research and policies aspects are identified as key priorities.  -There is no dedicated national budget for AMR research. |
