# Supplementary figures and images for: National action plans on antimicrobial resistance in Latin America: an analysis via a governance framework
Source: Health Policy Plan. 2024 Jan 5;39(2):188–97. doi: 10.1093/heapol/czad118 (PMC10883663; doi:10.1093/heapol/czad118)

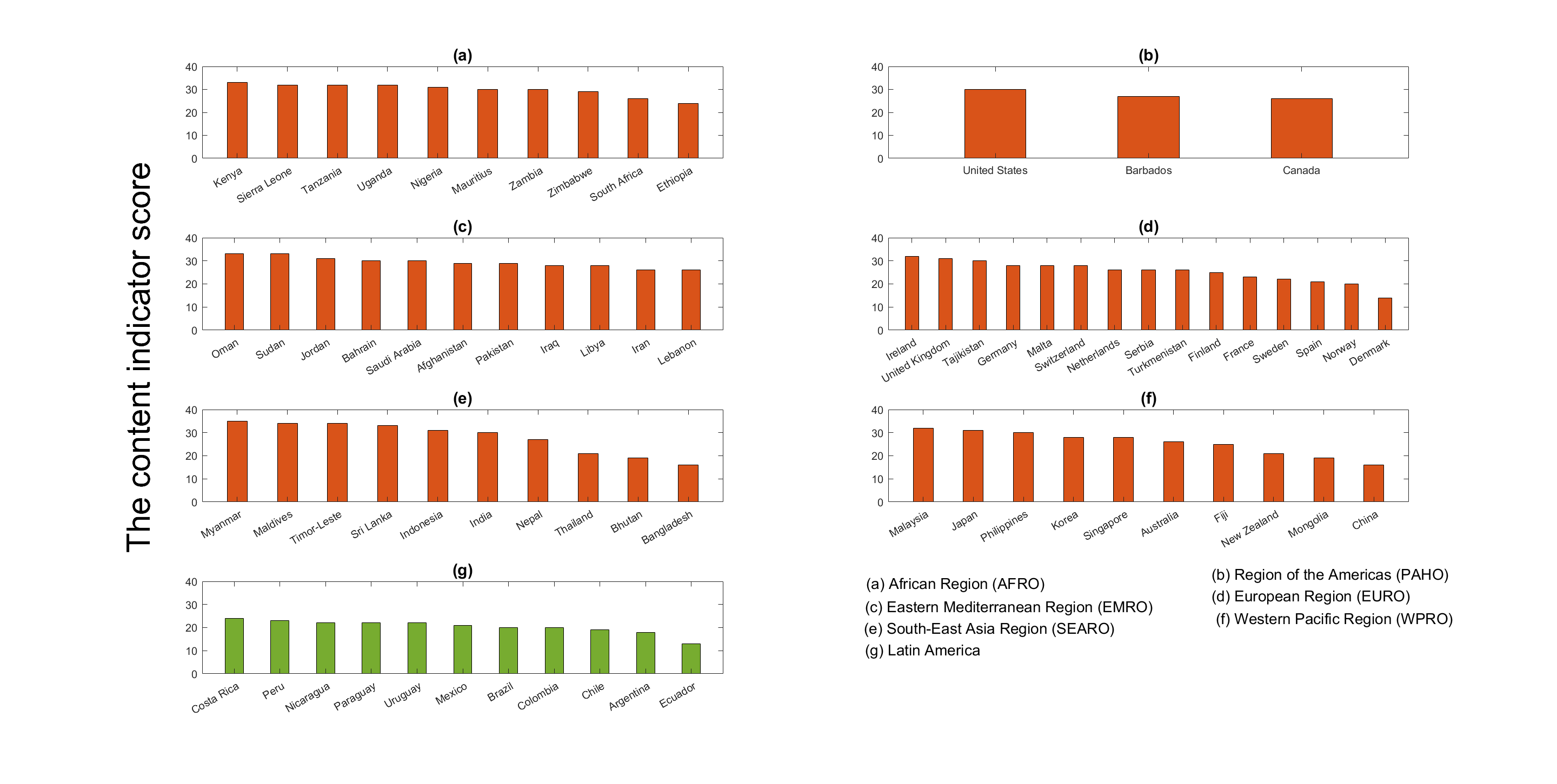

Supplement: czad118_Supp [file czad118_supp.zip › suppl_data/Figure S1_Supplementary material.tif]
